# The NPC1L1 Polymorphism 1679C>G Is Associated with Gallstone Disease in Chinese Patients

**DOI:** 10.1371/journal.pone.0147562

**Published:** 2016-01-22

**Authors:** Jian Wu, Wei Cui, Qu Cai, Jian Fei, Sheng-Dao Zhang, Tian-Quan Han, Hai Hu, Zhao-Yan Jiang

**Affiliations:** 1 Department of Hepatobiliary and Pancreatic Surgery, Shanghai East Hospital, Tongji University School of Medicine, Shanghai, China; 2 Department of Surgery, Shanghai Institute of Digestive Surgery, Ruijin Hospital, Shanghai Jiao Tong University School of Medicine, Shanghai, China; 3 Department of Surgery, Yijishan Hospital, Wannan Medical College, Jiangsu Province, China; University of Maryland, UNITED STATES

## Abstract

Niemann Pick Type C1 Like 1 (NPC1L1) protein plays a key role in intestinal and hepatic cholesterol metabolism in humans. Genetic variation in *NPC1L1* has been widely studied in recent years. We analyzed *NPC1L1* single nucleotide polymorphisms in Chinese gallstone disease patients to investigate their association with gallstone disease. *NPC1L1* mRNA expression was also measured in liver biopsies from patients with cholesterol gallstone disease and compared between genotypes. The G allele of the *g1679C>G (rs2072183)* polymorphism was significantly more prevalent in patients with gallstones compared with gallstone-free subjects. Moreover, patients carrying the G allele had lower hepatic *NPC1L1* mRNA expression and higher biliary cholesterol (molar percentages) and cholesterol saturation index. Our study suggests that the G allele of the *NPC1L1* polymorphism *g1679C>G* may be a positive marker of gallstone formation risk.

## Introduction

Niemann Pick Type C1 Like 1 (NPC1L1) is a putative protein involved in intestinal cholesterol absorption [1]. In humans, NPC1L1 is expressed in the liver, and localizes at hepatic canalicular membranes [2] where it facilitates biliary cholesterol uptake into hepatocytes and regulates biliary cholesterol content [3]. We recently reported that hepatic expression of *NPC1L1* was reduced in Chinese female patients with gallstone disease [4]. Moreover, another cohort study showed increased expression of *NPC1L1* in the jejunal mucosa from Chinese gallstone patients [5]. These data suggest a role for NPC1L1 in promoting gallstone formation by either increased uptake of luminal cholesterol into enterocytes or by decreased re-uptake of biliary cholesterol by hepatocytes.

Genetic variants of *NPC1L1* are reported to be associated with an altered rate of intestinal cholesterol absorption as measured by the ratio of plant sterol to lathosterol [6, 7], with changes in plasma low density lipoprotein cholesterol (LDL-C) levels [8], and with the different responses to ezetimibe [9–11], a drug targeting NPC1L1 [12]. However, little is known regarding the effect of *NPC1L1* variants on gallstone disease so far.

In this study, we genotyped *NPC1L1* SNPs in patients from two cohorts consisted of gallstone disease and their healthy counterparts, and compared *NPC1L1* expression in liver biopsies from gallstone patients as well. We found that the G allele of *g1679C>G (rs2072183)* is associated with gallstone disease. Moreover, patients carrying the G allele had lower hepatic *NPC1L1* mRNA expression and higher biliary cholesterol (molar percentages) and cholesterol saturation indices (CSIs).

## Materials and Methods

### Subjects

For the SNP association study, two cohorts were recruited, each consisting of gallstone disease (GS) patients and gallstone-free (GSF) subjects. In cohort I, 288 patients with cholesterol gallstone disease, who were subjected to cholecystectomy at the Shanghai Ruijin Hospital, and 272 gallstone-free subjects (served as controls) were recruited. In cohort II, 299 patients with cholesterol gallstone disease, who were subjected to cholecystectomy at the Shanghai East Hospital, and 249 gallstone-free subjects were recruited. The demographic characteristics in both cohorts were shown in S1 Table. Additionally, biopsies (0.1–0.5 g) were taken from the edge of the right liver lobe of cholesterol gallstone disease patients (n = 114), snap-frozen in liquid nitrogen, and later transferred and stored at −80°C for downstream analyses; gallbladder bile was also collected and stored at −80°C. For all subjects, 5 mL of venous blood was collected.

Gallstones were classified based on characteristic features of the cut surface and by enzymatic measurement of cholesterol content. Gallstones with cholesterol content > 50% were classified as cholesterol gallstones. All the gallstone-free subjects were either healthy volunteers underwent health examination in the hospital and were proved to have no gallstones by B-type ultrasonography (n = 256, m/f = 126/130 in cohort I and n = 237, m/f = 114/123 in cohort II) or patients underwent cholecystectomy because of gallbladder polyps or gallbladder adenomas who were confirmed to be gallstone free by dissection of the gallbladder in cases where patients underwent cholecystectomies because of gallbladder polyps or gallbladder adenomas (n = 16, m/f = 7/9 in cohort I and n = 12, m/f = 5/7 in cohort II). The study protocols were approved by the Ethical Committees both at Shanghai Ruijin Hospital and at Shanghai East Hospital and written informed consent was obtained from each patient.

### Genotyping

Genomic DNA was extracted from white blood cells using the QIAamp DNA blood mini kit (Qiagen, Hilden, Germany). Two SNP sites of the *NPC1L1* gene (*g-762T>C rs2073548* and *g1679C>G rs2072183*) were selected because the frequencies of all other sites were ≤ 2% in the Chinese population, as measured by genotyping 25 randomly selected control patients (S2 Table). The frequencies were similar to those reported by Chen et al. [13]. Genotyping was performed using Taqman allelic discrimination assays (Applied Biosystems, Foster City, CA, USA) on ABI 7900 (Applied Biosystems). Endpoint results were obtained by Sequence Detection System Software (Applied Biosystems).

### Relative mRNA quantification

Total hepatic RNA was extracted with TRIzol^®^ (Invitrogen, Carlsbad, CA, USA) and reverse transcribed into cDNA with the High-Capacity cDNA Reverse Transcript Kit (Applied Biosystems) and diluted 1:10 with DNase and RNase-free H_2_O. Real-time PCR was performed on an ABI 7900 using the SYBR Green assay (primer sequences were available on request) as described previously [4]. The delta-Ct method was used to calculate mRNA expression levels (expressed by ratio = 2^-deltaCt^ *100%), using Cyclophilin A as the control gene.

### Analysis of biliary lipids

Biliary cholesterol, bile acids, and phospholipids were extracted from gallbladder bile by FOLCH (chloroform/methanol = 2/1) and measured enzymatically as described [14]. CSIs were calculated using Carey’s critical table [15].

### Statistics

Qualitative data were compared using the Chi-square test. Quantitative data were expressed as means ± standard error of the mean and compared by analysis of variance. A post-hoc least significant difference test was performed to assess which genotypes were significantly different. The analysis was performed using SPSS 17.0 software (SPSS Inc., Chicago, IL, USA). Analysis of Hardy-Weinberg and haplotypes were performed by online SHEsis software (http://analysis.bio-x.cn/myAnalysis.php). Statistical significance was set at P<0.05.

## Results

### Distribution of genotypes and alleles between groups

In this study, we genotyped the *762T>C* and *1679C>G* loci of the *NPC1L1* gene. The allelic distributions of both loci were in line with Hardy–Weinberg disequilibrium (D’ value = 0.851, R^2^ value = 0.958). In both cohorts I and II, the minor allele frequency (MAF) of *g1679C>G (rs2072183)* was significantly higher in GS than in GSF as shown in Table 1. When the two cohorts were combined, the MAF of *g1679C>G (rs2072183)* was 38.8% in the GS group and 32.8% in the GSF group, respectively, P<0.01. The odds ratio (OR) for an association of the minor allele with gallstone disease was 1.30 (95% CI: 1.09–1.55, P<0.01) for all patients, and was 1.54 (95%CI: 1.19~1.99, P<0.01) in male and 1.12 (95%CI: 0.89~1.43, P>0.05) in female, respectively (S3 Table).

**Table 1:** Distribution of genotypes and alleles between groups.

		Cohort I		Cohort II		All			
		GSF(n = 272)	GS(n = 288)	GSF(n = 249)	GS(n = 299)	GSF(n = 521)	GS(n = 587)	OR(95%CI)	P
g-762T>C	TT	119	116	122	123	241	239		
rs2073548	TC	120	139	102	135	222	274		
	CC	33	33	25	41	58	74		
	MAF	34.2%	35.6%	30.5%	36.3%*	32.4%	35.9%	1.17(0.98~1.39)	0.08
g1679C>G	CC	125	100	117	118	242	218		
rs2072183	CG	109	146	107	136	216	282		
	GG	38	42	25	45	63	87		
	MAF	34%	39.9%*	31.50%	37.8%*	32.80%	38.8%**	1.30(1.09~1.55)	P<0.01

* P<0.05

** P<0.01, compared to GSF group

The frequency of MAF allele for g1679C>G was significantly higher in GS group than in GSF group in both cohorts.

The MAF of *g-762T>C (rs2073548)* was similar between GS and GSF in cohort I, but significantly higher in GS (36.3%) than in GSF (30.5%) in cohort II. When the two cohorts were combined, no significant difference in MAF between GS (35.9%) and GSF (32.4%) (Table 1) was present. The OR for an association of the minor allele with gallstone disease was 1.34 (95%: 1.03~1.74, P<0.05) in males and 1.04 (0.82~1.32) in females (S3 Table). Haplotype analysis showed that the C-G haplotype was associated with gallstone disease, with OR = 1.23 (95% CI: 1.02–1.47, P<0.05, Table 2).

**Table 2:** Haplotype frequencies between groups and OR associated with gallstone disease.

	GSF	GS	OR	95%CI	P
C-G	0.307	0.358	1.23	1.029–1.470	0.02
C-C	0.018	0.002	-	-	-
T-G	0.021	0.031	1.42	0.832–2.423	0.196
T-C	0.654	0.61	0.788	0.661–0.939	0.788

C-G represents for subjects carried C haplotype of g-762T>C polymorphism and G haplotype of g1679C>G polymorphism.

### Hepatic NPC1L1 expression and biliary lipid composition among genotypes

**Fig 1:**
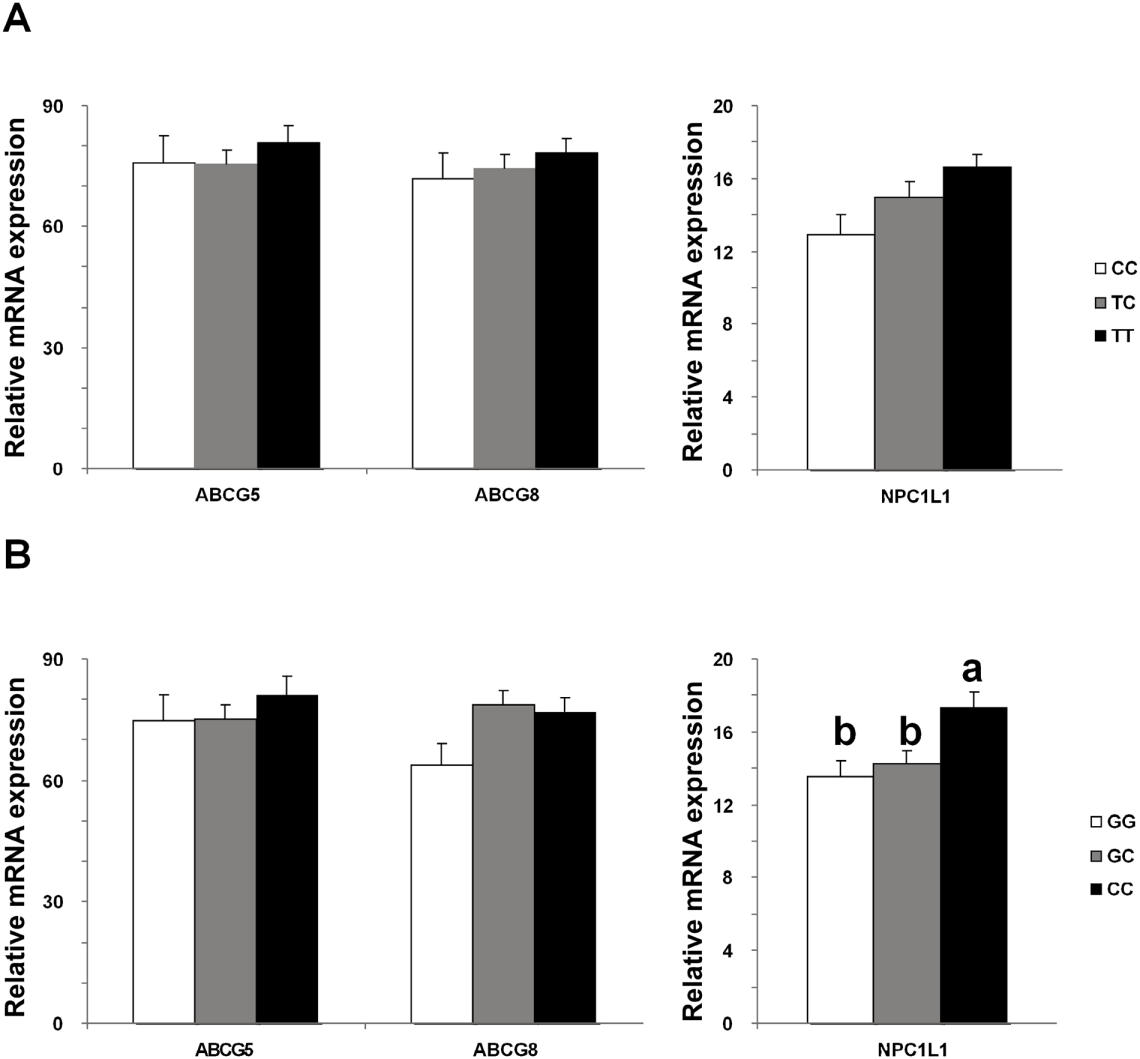
Comparison of mRNA expression of hepatic *ABCG5*, *ABCG8* and *NPC1L1* genes among genotypes of NPC1L1 gene polymorphisms. (A) g-762T>C polymorphism and (B) g1679C>G polymorphism. ‘a’ vs ‘b’: P<0.01 by ANOVA, post-hoc LSD analysis.

Patients carrying CC genotype of the g1679C>G loci had significantly higher hepatic *NPC1L1* mRNA expression than the GG or GC genotypes (P<0.05, Fig 1B), which was significant in females as well (P<0.05, S4 Table) and a similar trend in males (P = 0.109, S4 Table). No difference in *ABCG5* or *ABCG8* mRNA expression was found in either gender or all the subjects combined among genotypes (P>0.05, Fig 1B and S4 Table). In contrast, the biliary cholesterol molar percentages and CSIs were significantly higher in patients carrying the GG genotype (cholesterol molar percentage: 7.67±0.31; CSI: 1.06±0.04) and GC genotype (cholesterol molar percentage: 7.53±0.14; CSI: 1.06±0.02) than in patients carrying the CC genotype (cholesterol molar percentage: 6.52±0.22; CSI: 0.90±0.03, P<0.05, Table 3).

**Table 3:** Lipid composition in gallbladder bile between genotypes (means±S.E.M).

	g-762T>C			g1679C>G		
Genotypes	TT	TC	CC	CC	GC	GG
Cases	49	52	13	48	50	16
Chol%	6.30±0.25	6.67±0.76	6.42±0.59	6.52±0.22a	7.53±0.14b	7.67±0.31b
BA%	74.13±0.75	72.92±0.61	73.15±0.68	74.32±0.46	72.14±0.63	73.32±0.54
PL%	22.04±0.37	20.97±0.52	21.63±0.84	22.68±0.78	20.89±0.69	21.65±0.77
TL	13.28±0.46	12.53±0.76	12.68±0.53	12.37±0.73	12.52±0.81	12.73±0.27
CSI	0.97±0.03	1.03±0.03	0.98±0.04	0.90±0.03a	1.06±0.02b	1.06±0.04b

a vs. b, P<0.05 by ANOVA, post-hoc LSD analysis; Abbreviations: Chol: cholesterol; BA: bile acids; PL, phospholipids; TL, total lipid; CSI, cholesterol saturation index

There was no significant difference in either hepatic *NPC1L1* mRNA expression or biliary lipid composition among genotypes of the *g-762T>C* loci (Fig 1A and Table 3).

### Discussion

Cholesterol gallstone disease is a global problem that is influenced by several interacting genetic and environmental factors [16]. The pathogenesis of gallstone disease is complex involving imbalances in the secretion of biliary cholesterol and bile acids, crystallization/nucleation of cholesterol, impaired gallbladder and intestinal motility, and altered mucin secretion. NPC1L1 has a critical role in cholesterol metabolism and, although its genetic variation has been widely reported, little is known regarding the association of these variants with gallstone disease.

Here, we reported that the MAF of the *g1679C>G* loci of *NPC1L1* is higher in the GS than in the GSF group, as well as the C-G haplotype. Ours is the first study to suggest increased susceptibility to gallstone disease among Chinese individuals carrying the G allele of *g1679C>G*. During the preparation of this manuscript, inconsistence results concerning the association between the NPC1L1 common polymorphism with gallstone disease in European populations were reported [17–19]. Lauridsen et al genotyped NPC1L1 variants in 67,384 individuals from the general population in Copenhagen and found genotype score associated with a 22% increase in risk of symptomatic gallstone disease, especially in women [17]. However, such association could not be confirmed in studies by Ference et al (on European descents)[18], or Rodriguez et al (on women of European ancestry)[19], or when the three data were combined [20].

Unlike rodents, where *NPC1L1* is mainly expressed in the intestine, *NPC1L1* is highly expressed in human liver [1, 3, 21]. Hepatic *NPC1L1* has been demonstrated to play a direct role in regulating biliary cholesterol content [4, 22]. In our previous study, we found that patients with gallstone disease had decreased hepatic *NPC1L1* mRNA expression [4]. Owing to its role in biliary cholesterol re-uptake into hepatocytes, decreased hepatic *NPC1L1* expression may contribute to increased secretion of biliary cholesterol, which is the driving force of cholesterol precipitation [23, 24]. In line with its function in regulating biliary cholesterol levels, patients carrying the G allele of *g1679C>G* had decreased *NPC1L1* mRNA expression but increased biliary cholesterol content. These results suggest that subjects carrying the G allele of *g1679C>G* might be at a higher risk of biliary cholesterol supersaturation and gallstone formation. In line with these results, the MAF of *g1679C>G* was higher in GS than in GSF patients. The MAF of *g1679C>G* was 20–60% among different ethnicities (S5 Table). The overall MAF in our study was 36%, which is similar to two other studies with Chinese cohorts [13, 25], but different from Japanese [26, 27] and European cohorts [7, 11, 28]. As a synonymous SNP, the function of the *g1679C>G* polymorphism (*rs2072183*) is not fully understood. The mechanism whereby *rs2072183* variants influence cholesterol metabolism and response to ezetimibe needs further investigation.

In the intestine, *NPC1L1* is responsible for cholesterol absorption. However, we could not evaluate potential differences in intestinal cholesterol absorption in subjects with different *g1679C>G* alleles in this study. Indirect evidence––as indicated by the ratio of plant sterol to lathosterol, plasma LDL levels, and the response to ezetimibe––indicated that the G allele might be associated with enhanced cholesterol absorption. Zhao et al revealed that the G allele was associated with an augmented plant sterol induced cholesterol-lowering effect in hypercholesterolemic men [29]. Another study reported that carriers of the G allele had significantly greater concentrations of campesterol and sitosterol [7]. As plant sterol and lathosterol are generally considered surrogate markers for the efficiency of cholesterol absorption [30, 31], these two reports suggest that the G allele could enhance cholesterol absorption. In humans, LDL-C levels also reflect the efficiency of cholesterol absorption [32]. Polisecki et al. found that in Europeans, homozygous carriers of the minor alleles of four *NPC1L1* sites (*-18A>C*, *1679C>G*, *V1296V*, and *U3_28650A>G*) had 2–8% higher LDL-C levels and increased risk of coronary heart disease at baseline compared with homozygous carriers of the common alleles, owing to alterations in cholesterol absorption [8]. Miao et al suggested that the G allele contributes to increased serum total cholesterol and LDL-C levels in Han and Mulao Chinese male individuals, which also suggests increased cholesterol absorption. Regarding ezetimibe, G allele carriers exhibit an increased response to ezetimibe compared with carriers who are homozygous for the common allele [10, 11]. However, in our study, we did not find any difference in response to ezetimibe for the promoter of C allele comparing with T allele in Caco2 cells (S1 Fig). To date, no study has compared the difference in intestinal *NPC1L1* expression between genotypes. Because of the suggested role of NPC1L1 in uptaking biliary cholesterol into hepatocytes in human [3], there are concerns that ezetimibe treatment might induce gallstone formation through inhibition of hepatic NPC1L1. However, a short-term study showed no difference in gallstone occurrence after 6-month treatment of ezetimibe [33]. Very recently, the IMPROVE-IT study, after 6-year followed up, did not show increased incidence of gallstone disease by ezetimibe treatment [34] either. Furthermore, in a small group of gallstone patients, 30-day ezetimibe treatment seemed to reduce biliary cholesterol composition [35].

Interestingly, evidences regarding the potential effect of the *g1679C>G* polymorphism are inconsistent. Lupattelli et al. [7] found that the G allele was associated with significantly greater concentrations of campesterol and sitosterol, but not lathosterol. Meanwhile, Maeda et al. [27] reported that only campesterol levels differ between genotypes. Similar to plant sterol and lathosterol, LDL-C was reported to be increased in G carriers by Polisecki et al. [8] and Miao et al. [25] but not in three other studies [7, 13, 27]. These conflicting results can probably be attributed to differences in study design, sample size, ethnicity, interaction with other SNPs, and gene–environment interaction.

There are some limitations in the present study. First, protein levels of hepatic genes could not be measured due to limited biopsy samples. Second, although difference in biliary cholesterol saturation was found between genotypes, we should be aware that the gallbladder bile might not reflect the hepatic secretion of cholesterol because bile composition can be concentrated and modified by epithelium in gallbladder.

### Conclusion

The most notable finding of our study is the significantly higher MAF of *g1679C>G* in GS patients compared with GSF individuals. Moreover, the minor allele of the *NPC1L1 g1679C>G* loci was associated with lower hepatic *NPC1L1* mRNA expression and higher biliary molar percentages of cholesterol and CSIs. Collectively, these data suggest that the G allele of the *NPC1L1 g1679C>G* loci may be a risk factor for gallstone disease.

## Supporting Information

S1 Fig Effect of simvastatin and ezetimibe on *NPC1L1* promoter activity in Caco2. (PDF)

S1 Table Demographic characteristic of patients with and without gallstone disease in the two cohorts. (DOCX)

S2 Table Allele frequencies of SNPs detected in the preliminary study. (DOCX)

S3 Table Distribution of genotype and allele frequency between GSF and GS groups in males and females. (DOCX)

S4 Table Comparison of mRNA expression of hepatic genes between genotypes in females and males. (DOCX)

S5 Table Genotype frequency of g1679C>G in different populations. (DOCX)

## Abbreviations

BA: bile acids
Chol: cholesterol
CSI: cholesterol saturation index
GS: gallstone disease
GSF: gallstone-free
HNFα: hepatic nuclear factor α
LDL-C: low-density lipoprotein cholesterol
LXR: liver X receptors
MAF: minor allele frequency
NPC1L1: Niemann Pick Type C1 Like 1
OR: odds ratio
SNPs: single nucleotide polymorphisms
SREBP2: sterol regulatory element binding protein 2
TL: total lipid
