# Supplementary material for: The NPC1L1 Polymorphism 1679C>G Is Associated with Gallstone Disease in Chinese Patients
Source: PLoS One. 2016 Jan 22;11(1):e0147562. doi: 10.1371/journal.pone.0147562 (PMC4723254; doi:10.1371/journal.pone.0147562)
Supplement: S1 Fig — (PDF) [file pone.0147562.s001.pdf]

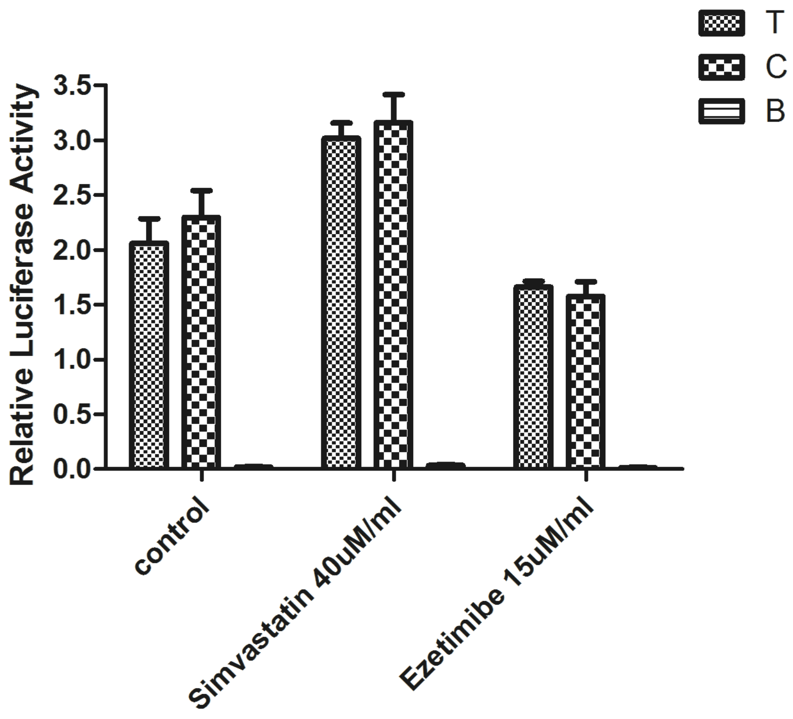

Figure S1 Effect of simvastatin and ezetimibe on *NPC1L1* promoter activity in Caco2.

Compared with normal group, the promoter activity of ezetimibe (15uM/ml) was markedly inhibited in -762T (normal = 2.060±0.224 vs ezetimibe = 1.660±0.055, $P<0.05$) and -762C (normal = 2.296±0.245 vs ezetimibe = 1.575±0.137, $P<0.05$). When simvastatin (40uM/ml) added, the promoter activity in both -762T (normal = 2.060±0.224 vs simvastatin = 3.061±0.142, $P<0.05$) and -762C (normal = 2.296±0.245 vs simvastatin = 3.161±0.256, $P<0.05$) showed a notable increasing comparing the normal group ($P<0.05$). No significant difference of the promoter activity was found between -762T and -762C in three groups ($P<0.05$).
