# Supplementary material for: The NPC1L1 Polymorphism 1679C>G Is Associated with Gallstone Disease in Chinese Patients
Source: PLoS One. 2016 Jan 22;11(1):e0147562. doi: 10.1371/journal.pone.0147562 (PMC4723254; doi:10.1371/journal.pone.0147562)
Supplement: S1 Table — (DOCX) [file pone.0147562.s002.docx]

**S1 Table Demographic characteristic of patients with and without gallstone disease in the two cohorts (means** ± **SEM)**

|  | Cohort I | | Cohort II | |
| --- | --- | --- | --- | --- |
|  | GSF | GS | GSF | GS |
| Case(male/female) | 272(133/139) | 288(129/159) | 249(119/130) | 299(130/169) |
| Age(year) | 53.7±0.66 | 52.2±0.88 | 49.9±0.87 | 53.41±0.82 |
| BMI(kg/m^2^) | 22.39±0.11 | 22.61±0.14 | 23.06±0.28 | 24.15±0.24 |
| TC(mmol/L) | 4.08±0.12 | 4.14±0.09 | 4.42±0.09 | 4.58±0.19 |
| TG(mmol/L) | 1.60±0.05 | 1.61±0.07 | 1.62±0.10 | 1.39±0.06 |
| HDL(mmol/L) | 1.19±0.03 | 1.21±0.02 | 1.27±0.03 | 1.32±0.05 |
| LDL(mmol/L) | 2.28±0.12 | 2.21±0.09 | 2.48±0.09 | 3.00±0.09 |
