# Supplementary material for: The NPC1L1 Polymorphism 1679C>G Is Associated with Gallstone Disease in Chinese Patients
Source: PLoS One. 2016 Jan 22;11(1):e0147562. doi: 10.1371/journal.pone.0147562 (PMC4723254; doi:10.1371/journal.pone.0147562)
Supplement: S2 Table — (DOCX) [file pone.0147562.s003.docx]

**S2 Table Allele frequencies of SNPs detected in the preliminary study**

|  | | | | |
| --- | --- | --- | --- | --- |
| sample number | rs2073548 | rs17655652 | rs41279633 | rs2072183 |
|  | -762T>C | -133C>T | -18G>T | 1679C>G |
| 19 | CT | TT | GG | CG |
| 20 | CT | TT | GG | CG |
| 22 | CT | TT | GG | CG |
| 26 | CT | TT | TG | GG |
| 28 | CC | TT | GG | GG |
| 29 | TT | TT | GG | CC |
| 31 | CC | TT | GG | GG |
| 32 | CT | TT | GG | CG |
| 35 | CT | TT | GG | CG |
| 39 | CT | TT | GG | CG |
| 42 | CT | TT | GG | CG |
| 45 | CT | TT | GG | CG |
| 49 | CT | TT | GG | CG |
| 52 | TT | TT | GG | CC |
| 54 | TT | TT | GG | CC |
| 58 | TT | TT | GG | CC |
| 61 | TT | TT | GG | CC |
| 63 | TT | TT | GG | CC |
| 64 | CC | TT | GG | GG |
| 65 | CT | CT | GG | CG |
| 66 | TT | TT | GG | CC |
| 67 | TT | TT | GG | CC |
| 68 | CT | TT | GG | CG |
| 69 | TT | TT | GG | CC |
| 71 | TT | TT | GG | CC |
| allele | T=64% | T=98% | G=98% | C=62% |
|  | C=36% | C=2% | T=2% | G=38% |
| genotype | CT=48% | TT=96% | GG=96% | CG=44% |
|  | TT=40% | CT=4% | TG=4% | CC=40% |
|  | CC=12% | CC=0% | TT=0% | GG=16% |
