# Supplementary material for: The NPC1L1 Polymorphism 1679C>G Is Associated with Gallstone Disease in Chinese Patients
Source: PLoS One. 2016 Jan 22;11(1):e0147562. doi: 10.1371/journal.pone.0147562 (PMC4723254; doi:10.1371/journal.pone.0147562)
Supplement: S3 Table — (DOCX) [file pone.0147562.s004.docx]

**S3 Table Distribution of genotype and allele frequency between GSF and GS groups in males and females**

|  |  | Male | |  | Female |  |  |
| --- | --- | --- | --- | --- | --- | --- | --- |
|  |  | GSF | GS | OR (95%CI) | GSF | GS | OR (95%CI) |
| -762 | TT | 129 | 99 |  | 112 | 140 |  |
|  | TC | 93 | 129 |  | 129 | 145 |  |
|  | CC | 30 | 31 |  | 28 | 43 |  |
|  | MAF% | 30.36 | 36.87* | 1.34 (1.03~1.74) | 34.38 | 35.21 | 1.04 (0.82~1.32) |
|  |  |  |  |  |  |  |  |
| 1679 | CC | 126 | 86 |  | 116 | 132 |  |
|  | GC | 94 | 132 |  | 122 | 150 |  |
|  | GG | 32 | 41 |  | 31 | 46 |  |
|  | MAF% | 31.35 | 41.32** | 1.54 (1.19~1.99) | 34.20 | 36.89 | 1.12 (0.89~1.43) |

* P<0.05, **P<0.01**S**
