# Supplementary material for: The NPC1L1 Polymorphism 1679C>G Is Associated with Gallstone Disease in Chinese Patients
Source: PLoS One. 2016 Jan 22;11(1):e0147562. doi: 10.1371/journal.pone.0147562 (PMC4723254; doi:10.1371/journal.pone.0147562)
Supplement: S4 Table — (DOCX) [file pone.0147562.s005.docx]

**S4 Table Comparison of mRNA expression of hepatic genes between genotypes in females and males (means** ± SEM)

|  |  | Female |  |  |  |  | Male |  |  |  |
| --- | --- | --- | --- | --- | --- | --- | --- | --- | --- | --- |
|  |  | number | ABCG5 | ABCG8 | NPC1L1 |  | number | ABCG5 | ABCG8 | NPC1L1 |
| -762T>C | CC | 4 | 65.3±7.8 | 67.0±10.0 | 10.7±1.6 |  | 9 | 80.6±8.5 | 73.8±8.8 | 14.0±1.2a |
|  | TC | 32 | 74.4±4.6 | 79.5±4.7 | 15.8±1.2 |  | 20 | 76.7±5.7 | 66.4±5.0 | 13.6±1.1a |
|  | TT | 33 | 88.3±5.6 | 84.5±4.8 | 15.8±0.9 |  | 16 | 64.8±4.6 | 64.9±5.2 | 18.4±1.1b |
|  |  |  |  |  |  |  |  |  |  |  |
| 1679C>G | GG | 6 | 62.2±6.7 | 63.7±7.5 | 11.9±0.9a |  | 10 | 82.4±8.4 | 63.8±7.8 | 14.6±1.2 |
|  | GC | 31 | 76.5±4.6 | 85.6±5.1 | 14.3±1.0a |  | 19 | 72.9±5.6 | 66.9±4.7 | 14.1±1.2 |
|  | CC | 32 | 87.8±5.8 | 80. 2±4.5 | 17.2±1.2b |  | 16 | 68.0±5.0 | 70.1±6.2 | 17.5±1.2 |

a vs b P<0.05 by ANOVA
