# Supplementary material for: The NPC1L1 Polymorphism 1679C>G Is Associated with Gallstone Disease in Chinese Patients
Source: PLoS One. 2016 Jan 22;11(1):e0147562. doi: 10.1371/journal.pone.0147562 (PMC4723254; doi:10.1371/journal.pone.0147562)
Supplement: S5 Table — (DOCX) [file pone.0147562.s006.docx]

**S5 Table Genotype frequency of g1679C>G in different populations**

|  | | | | | | | |
| --- | --- | --- | --- | --- | --- | --- | --- |
| Genotype frequency of g1679C>G | | | | | | | |
|  | |  |  | C/C | C/G | G/G | MAF% |
| This study | GSF | Chinese | Asian | 0.46(242) | 0.41(212) | 0.13(63) | 32.8 |
| This study | GS | Chinese | Asian | 0.37(218) | 0.48(282) | 0.15(87) | 38.8 |
| Chen CW et al^[1]^ | normocholesterolemic | Chinese | Asian | 0.40(77) | 0.51(97) | 0.09(18) | 34.6 |
|  | dyslipidemia | Chinese | Asian | 0.34(11) | 0.47(15) | 0.19(6) | 42.2 |
| Miao L et al^[2]^ | normocholesterolemic | Chinese(Han) | Asian | 0.39(290) | 0.47(346) | 0.14(102) | 37.3 |
|  | normocholesterolemic | Chinese(Mulao) | Asian | 0.50(343) | 0.41(281) | 0.09(64) | 29.7 |
| Kashiwabara Y et al^[3]^ | dyslipidemia | Japanese | Asian | 0.15(17) | 0.49(57) | 0.36(41) | 60.4 |
| Maeda T et al^[4]^ | normocholesterolemic | Japanese | Asian | 0.35(50) | 0.46(65) | 0.19(27) | 41.9 |
| Lupattelli G et al^[5]^ | dyslipidemia | Italian | Caucasian | 0.56(49) | 0.41(36) | 0.02(2) | 23.0 |
| Pisciotta L et al^[6]^ | dyslipidemia | Italian | Caucasian | 0.63(44) | 0.33(23) | 0.04(3) | 20.7 |
| Martín B et al^[7]^ | normocholesterolemic | Spanish | Caucasian | 0.57(155) | 0.39(107) | 0.04(11) | 23.6 |
|  | dyslipidemia | Spanish | Caucasian | 0.54(146) | 0.40(109) | 0.06(17) | 26.3 |
| a, the genotype frequeny of 1679C>G is significant different between Chinese to Janpanese, Italian, Spanish respectively(P<0.001) | | | | | | |  |
| b, the genotype frequeny of 1679C>G is significant different between Janpanese to Italian, Spanish respectively(P<0.001) | | | | | | |  |
| c, no significant different between Italian to Spanish(P<0.001) | | | | | | |  |

**References**

[1] Chen CW, Hwang JJ, Tsai CT, Su YN, Hsueh CH, Shen MJ, et al. The g.-762T>C polymorphism of the NPC1L1 gene is common in Chinese and contributes to a higher promoter activity and higher serum cholesterol levels. Journal of human genetics. 2009;54(4):242-7.

[2] Miao L, Yin RX, Hu XJ, Wu DF, Cao XL, Li Q, et al. Association of rs2072183 SNP and serum lipid levels in the Mulao and Han populations. Lipids in health and disease. 2012;11:61.

[3] Kashiwabara Y, Kobayashi Y, Koba S, Kohyama N, Ohbayashi M, Murayama JI, et al. Gene polymorphism and frequencies of the NPC1L1 Gene (rs2072183, rs217434 and rs217428) in Japanese patients with dyslipidemia. Journal of clinical pharmacy and therapeutics. 2014;39(5):551-4.

[4] Maeda T, Honda A, Ishikawa T, Kinoshita M, Mashimo Y, Takeoka Y, et al. A SNP of NPC1L1 affects cholesterol absorption in Japanese. Journal of atherosclerosis and thrombosis. 2010;17(4):356-60.

[5] Lupattelli G, Pisciotta L, De Vuono S, Siepi D, Bellocchio A, Melis F, et al. A silent mutation of Niemann-Pick C1-like 1 and apolipoprotein E4 modulate cholesterol absorption in primary hyperlipidemias. Journal of clinical lipidology. 2013;7(2):147-52.

[6] Pisciotta L, Bellocchio A, Bertolini S. Nutraceutical pill containing berberine versus ezetimibe on plasma lipid pattern in hypercholesterolemic subjects and its additive effect in patients with familial hypercholesterolemia on stable cholesterol-lowering treatment. Lipids in health and disease. 2012;11:123.

[7] Martin B, Solanas-Barca M, Garcia-Otin AL, Pampin S, Cofan M, Ros E, et al. An NPC1L1 gene promoter variant is associated with autosomal dominant hypercholesterolemia. Nutrition, metabolism, and cardiovascular diseases : NMCD. 2010;20(4):236-42.
